# Clinical brain PET research must embrace multi-centre collaboration and data sharing or risk its demise

**DOI:** 10.1007/s00259-019-04541-y

**Published:** 2019-11-12

**Authors:** Granville James Matheson, Pontus Plavén-Sigray, Jouni Tuisku, Juha Rinne, David Matuskey, Simon Cervenka

**Affiliations:** 1 Centre for Psychiatry Research, Department of Clinical Neuroscience, Karolinska Institutet and Stockholm County, Stockholm, Sweden; 2 Turku PET Centre, University of Turku, Turku, Finland; 3 Turku PET Centre, Turku University Hospital, Turku, Finland; 4 PET Center, Department of Radiology and Biomedical Imaging, Yale University, New Haven, USA; 5 Department of Psychiatry, Yale University, New Haven, USA

Dear Editor,

Genetics in the early 2000s consisted primarily of studies in small samples from individual research centres. Following the successful initial identification of very rare genetic variants which cause large effects, the search continued for individual genes which might explain a substantial proportion of the phenotypic variance in the wider population. However, it soon became clear that such genes simply do not exist, and that nearly all conclusions of the latter studies were incorrect [1]. To rise to the challenge, the field collectively moved towards collaborative research, yielding multi-centre sample sizes of up to tens of thousands, and genetics is now widely considered to produce robust scientific results [2]. During recent years, researchers within several fields of neuroimaging research, particularly MRI and fMRI, have begun to make this transition to data sharing and collaborative research, facilitated by technical developments in data handling and analysis [3–5].

Just like genetics, clinical positron emission tomography (PET) research has provided answers to numerous research questions across several domains. For example, the dopamine transporter shows clear decreases in Parkinson’s disease, dopamine synthesis capacity is increased in schizophrenia, and mony brain neurotransmission proteins show distinct decreases across the lifespan. However, for the well-established tracers and targets, it can be argued that we have already picked most of the low-hanging fruit. In the continued quest to break new ground, it is likely that most of the studied effects will be small, which means that large sample sizes will be needed to reach the threshold of statistical significance [6]. If we instead continue to use small sample sizes to search for subtle true effects, we run the risk of fooling ourselves into seeing “patterns” in what is really noise, leading to reporting of spurious effects. Further, with small samples, even when we correctly identify true effects as significant, our effect size estimates will be biased upward [6, 7]. We acknowledge that there will always be an important role for small exploratory studies for generating new hypotheses, but the subsequent confirmation and quantitative description of these hypotheses simply requires higher standards of evidence to move the field forward. Importantly, the problem of unreliable findings is by no means restricted to PET research, as has recently been evidenced by “replication crises” in other fields such as psychology, economics, and preclinical drug discovery research [8–12].

Unfortunately, large samples in the field of PET are unattainable for many individual research centres, owing to the high cost and technical difficulty of the method [13]. Traditionally, the proposed remedy to the issue of small sample size studies has been to perform meta-analyses to gain an overall, field-wide estimate of the studied effect. However, traditional meta-analysis has its own set of limitations. If the individual studies entered into a meta-analysis consist of biased effect size estimates, then the overall effect size will also be misleading [14–16]. It is also not possible to control for confounders, or to account for differences in outcome measures between studies [17]. One solution is to instead make use of the original data points collected by individual research centres. In the previous issue of EJNMMI, we report the results of such a multi-centre collaboration, or “mega-analysis” (Tuisku et al. [18]). By synthesising translocator protein (TSPO)–binding data from three different centres, effects were shown for age, BMI, and sex on TSPO, some of which were not evident in previous studies using smaller samples. Apart from informing the design and interpretation of TSPO PET studies, the results may also open up new avenues of research into the biological role of TSPO.

Multi-centre collaboration and data sharing entail certain considerations. With more researchers working on the same problem, the risk for differences in opinion regarding outcome measures, statistical analyses, and even the nature of the hypotheses increases [19]. We have found it useful to formally make these decisions in advance. A Memorandum of Understanding (MoU) may serve as an initial step, containing rules regarding data handling, the general outline of the analysis, as well as author number and order. This document can then be complemented by a specific pre-registration protocol for the analysis, detailing how data will be synthesised, which hypotheses will be tested, which statistical models will be used to make inference, etc. [19, 20]. When all authors have come to an agreement on the content, the protocol can be locked and uploaded to a date-stamped public repository. In the ensuing analysis, deviations from the pre-registration are still possible, provided that they are reported in addition to the original protocol.

Importantly, sharing of individual participant outcome measures, such as binding values, is only the first step. By using data in as raw a form as possible, the data processing in the combined analysis can be made more homogeneous. This can be achieved by using either a centralised analysis, or by using reproducible, open-source tools for which all procedures are scripted and can be run in an identical fashion [21, 22]. Hence, in the case of PET studies, the sharing of time activity curves is better than sharing of binding outcomes, while raw image data is better still, allowing for homogeneous data analysis all the way from image processing to pharmacokinetic modelling [23]. An additional measure to minimise between-centre differences would then be to use harmonised protocols for data collection.

The sharing of raw image data has historically been challenging, as storage and processing of files can differ between, or even within research groups. These complications are effectively resolved by the recently developed Brain Imaging Data Structure (BIDS) [3]. BIDS consists of a set of standards for storing brain imaging data, such that preprocessing and analysis can be performed in a standardised fashion, further simplified by BIDS Apps [4]. Further, the OpenNeuro repository allows for *open sharing* of neuroimaging data according to the BIDS standard, and is already in wide use by the MRI, EEG, and MEG research communities. Today, there are also a limited number of PET measurements available on this platform (e.g. https://openneuro.org/datasets/ds001421/versions/1.0.1). At the NeuroReceptor Mapping conference in London 2018, a proposal to set up an open PET data sharing archive using the BIDS standard received unanimous support.

By sharing individual participant data, regulatory aspects regarding data integrity come into play. The principle for data sharing adopted by the EU commission is that of “as open as possible, as closed as necessary” [24]. Contrary to common belief, the General Data Protection Regulation (GDPR) is designed to *facilitate* sharing of research data and collaboration, provided that sufficient steps have been taken to perform de-identification. A full interpretation of the implications of this new legislation is currently underway for many research centres/countries, and at present local guidelines may differ. Either way, we encourage researchers to begin as early as possible to ask research participants for permission for open sharing of research data for ongoing and planned PET studies, in order to ensure that future legal obstacles can be minimised. Efforts are underway to assist researchers in this matter, by creating template forms for informed consent which comply with all regulatory statutes (https://open-brain-consent.readthedocs.io).

Within the PET brain imaging field, we are now at a crossroads. Will we continue to work solely within individual research centres, using small samples to yield incomplete, or even misleading, results from confirmatory studies; or will we make the transition to multi-centre collaboration and data sharing as exemplified by the genetics community? We hope for the latter, sooner rather than later, in order to ensure the continued success of PET research in driving our understanding of the biochemical basis of brain function and dysfunction.
